# Energy transition pathways amongst low-income urban households: A mixed method clustering approach

**DOI:** 10.1016/j.mex.2021.101491

**Published:** 2021-08-14

**Authors:** André P. Neto-Bradley, Rishika Rangarajan, Ruchi Choudhary, Amir B. Bazaz

**Affiliations:** a Department of Engineering, University of Cambridge, UK; b Indian Institute for Human Settlements, India; c Data Centric Engineering, Alan Turing Institute, UK

**Keywords:** Mixed methods, Clustering, Data science, Energy access

## Abstract

Studies on clean energy transition amongst low-income urban households in the Global South use an array of qualitative and quantitative methods. However, attempts to combine qualitative and quantitative methods are rare and there are a lack of systematic approaches to this. This paper demonstrates a two stage approach using clustering methods to analyse a mixed dataset containing quantitative household survey data and qualitative interview data. By clustering the quantitative and qualitative data separately, latent groups with common characteristics and narratives arising from each of the two analyses are identified. A second stage of clustering identifies links between these qualitative and quantitative clusters and enables inference of energy transition pathways followed by low-income urban households defined by both quantitative characteristics and qualitative narratives. This approach can support interdisciplinary collaboration in energy research, providing a systematic approach to comparing and identifying links between quantitative and qualitative findings.

• A mixed dataset comprising of quantitative survey data and qualitative interview data on low-income household energy use is analysed using hierarchical clustering to detect communities within each dataset.
• Interviewees are matched to quantitative survey clusters and a second stage of clustering is performed using cluster membership as variables.
• Second stage clusters identify common pairs of survey and interview clusters which define energy transition pathways based on socio-economic characteristics, energy use patterns, and narratives for decision making and practices.

## Graphical abstract

**Figure:**
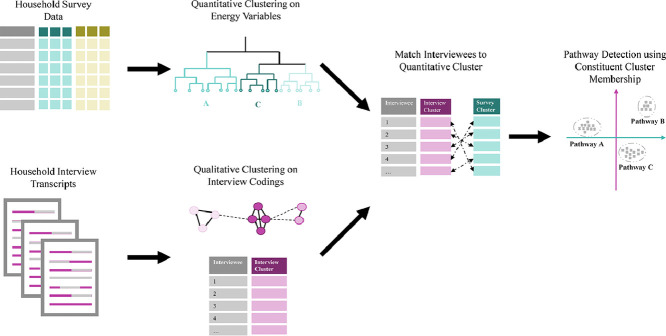

Specifications table

**Table:** 

Subject Area:	Energy
More specific subject area:	*Characterisation of energy access barriers and transition pathways amongst low-income urban households*
Method name:	*Mixed method cluster analysis*
Name and reference of original methods:	*Hierarchical Clustering and Qualitative Data Analysis* *Ward, J.H., 1963. Hierarchical Grouping to Optimize an Objective Function. J. Am. Stat. Assoc. 58, 236–244.*10.1080/01621459.1963.10500845 *Fujita, A., Takahashi, D.Y., Patriota, A.G., 2014. A non-parametric method to estimate the number of clusters. Comput. Stat. Data Anal. 73, 27–39*. 10.1016/j.csda.2013.11.012 *Gower, J.C., 1971. A General Coefficient of Similarity and Some of Its Properties. Biometrics 27, 857–871.* *Bansal, N., Blum, A., Chawla, S., 2004. Correlation Clustering. Mach. Learn. 56, 89–113*. 10.1023/B:MACH.0000033116.57574.95
Resource availability:	*An anonymized sample dataset for our case study is available along with sample code than can be used to carry out key steps of our method using this dataset. Data available at:*10.17863/CAM.59870

*Method details

## Introduction

Studies on drivers of clean energy transitions and issues of energy access amongst low-income households in the Global South typically make use of either purely quantitative methods (such as regression analysis), or qualitative methods (such as in-depth interviews), to the exclusion of the other. However, energy access and the practices and decisions around a household's energy use involve a complex interaction of social, economic, cultural, and community features which can only be understood through both qualitative and quantitative data and methods. This paper proposes a simple yet powerful approach combining qualitative data analysis with statistical clustering to identify links between qualitative information and quantitative data and thus infer different energy transition pathways followed by low-income urban households.

This method is motivated by the need to address the challenge of bridging disciplinary divides in residential energy research. As Sovacool et al. [47] elaborate there are a wide range of research methods, both quantitative and qualitative, used by different disciplines to study energy use. Qualitative social science approaches can offer great explanatory power and rich detail, but results do not lend themselves to scaling. In contrast quantitative approaches are better suited to identifying trends at scale but often do so at the cost of explanatory power. The differing ontological assumptions of these approaches and the disciplines that use them can make direct integration of methods difficult [22]. Instead there is a need to bridge across disciplines and approaches with common framing, and sharing of data in an iterative process [49]. Clustering methods have been shown to offer insight into residential energy transitions in India, characterizing heterogeneity across users of particular fuels and technologies [40]. This method proposes using clustering methods to integrate qualitative and quantitative approaches to characterize heterogeneity amongst households in energy transitions, offering a means to bridge different disciplines.

Clustering methods are concerned with finding groups of similar instances within a dataset, optimizing for similarity between instances in the cluster and dissimilarity between clusters [44]. There are different ways of identifying such latent groups in data broadly classified as either approaches focused on partition of data or model based approaches [30]. There is a substantial body of literature on clustering methods for mixed data, looking at how different datatypes such as categorical and numerical data, which are common in socio-economic datasets, can be jointly clustered. A longstanding and popular method involves the use of the Gower similarity measure with hierarchical clustering [25], although many approaches to using mixed data often involve coding or discretisation of data [16]. The *k*-prototypes method expands the *k*-means clustering method to include mixed datatypes [32], however this requires user specified weighting of different datatypes. More recent approaches have sought to offer methods that ensure equitable weighting to different datatypes and do not require conversion or coding of data, although implementation of such methods can be more involved [13,15]. Selection of appropriate clustering methods is difficult and as discussed by Hennig [29] when using real world social data latent groups are not necessarily clear cut, and choice of clustering method is highly context dependent. Another important distinction in clustering methods is between those that can be described as ‘crisp’ which assign an instance to a single cluster, versus those that are ‘fuzzy’ and quantify degrees of uncertainty in assignment of an instance to a cluster [12]. Research into applications of fuzzy clustering and how it can handle uncertainty in data shows how this can be particularly useful in a decision-making context, although implementation and interpretation can be less straightforward.

**Fig. 1:**
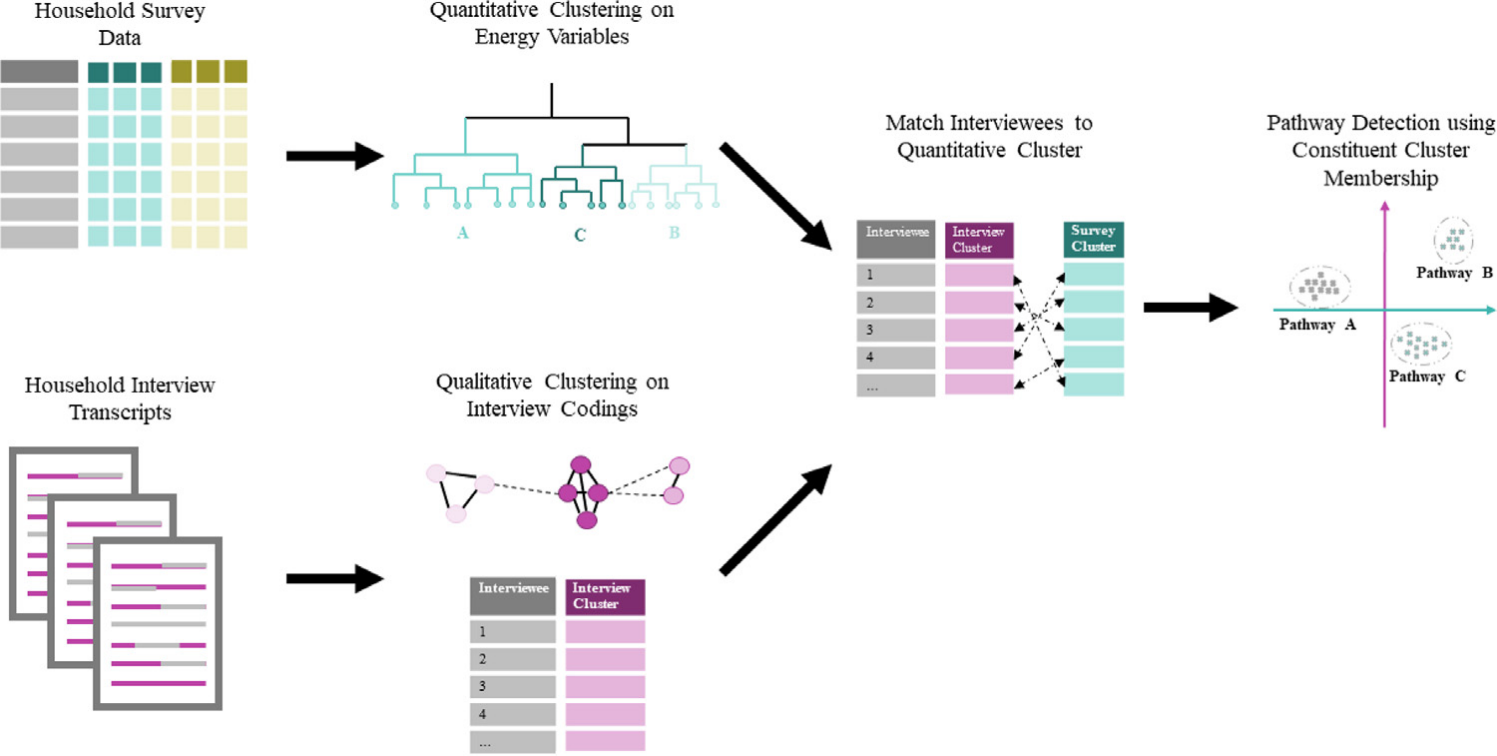
Schematic overview of mixed method cluster analysis. The analysis starts with a first stage clustering of both the quantitative survey data and the qualitative interview transcripts. Hierarchical clustering methods are used in this first stage, with the interviews coded for the qualitative clustering. The interview respondents are then matched to a corresponding survey cluster based on common socio-economic variables and a second stage of clustering is performed to identify distinct combinations of qualitative and quantitative clusters that characterise different energy transition pathways.

A relatively simple approach is taken in the method proposed, using hierarchical ‘crisp’ clustering that aims to allow separate qualitative and quantitative analysis and links these to identify likely transition pathways. The simplicity and clarity is motivated by the need to facilitate comprehension regardless of familiarity with quantitative methods, with a view to enabling interdisciplinary collaboration. This method involves two separate stages of community detection using two datasets collected from the same geographic area. The schematic in Fig. 1 provides an overview of the method which uses both a quantitative dataset containing household level socio-economic and energy use data, and a second qualitative dataset consisting of semi-structured interviews on household energy practices and decision making. Each dataset is clustered to identify common groups, and then the interview respondents are matched to a quantitative survey cluster. A second stage of clustering is performed on the quantitative and qualitative cluster membership of the interview respondents to identify the distinct energy transition pathways amongst these households, defined by socio-economic and energy use characteristics and associated narratives.

This method requires a dataset consisting of quantitative survey data and in-depth qualitative interviews collected from the same geographic area of interest. A protocol for data collection is provided under additional information but it should be noted that the mixed methods clustering could be applied to data collected by other means so long as it satisfies the requirements of the analysis. Throughout this paper aspects of the method are demonstrated through an example dataset on low-income urban households in Bangalore, India. The detailed analysis and inference from this dataset are presented in a separate paper by Neto-Bradley et al. [41]. The remainder of this paper begins by describing the two stages of analysis for the proposed methodology, before considering the benefits and limitations of the method using results and outputs for the example case of Bangalore. The paper finishes with concluding remarks on the method as well as a brief discussion on possible avenues for further improvement.

## Mixed data pathway clustering

### First step clustering analysis

**Fig. 2:**
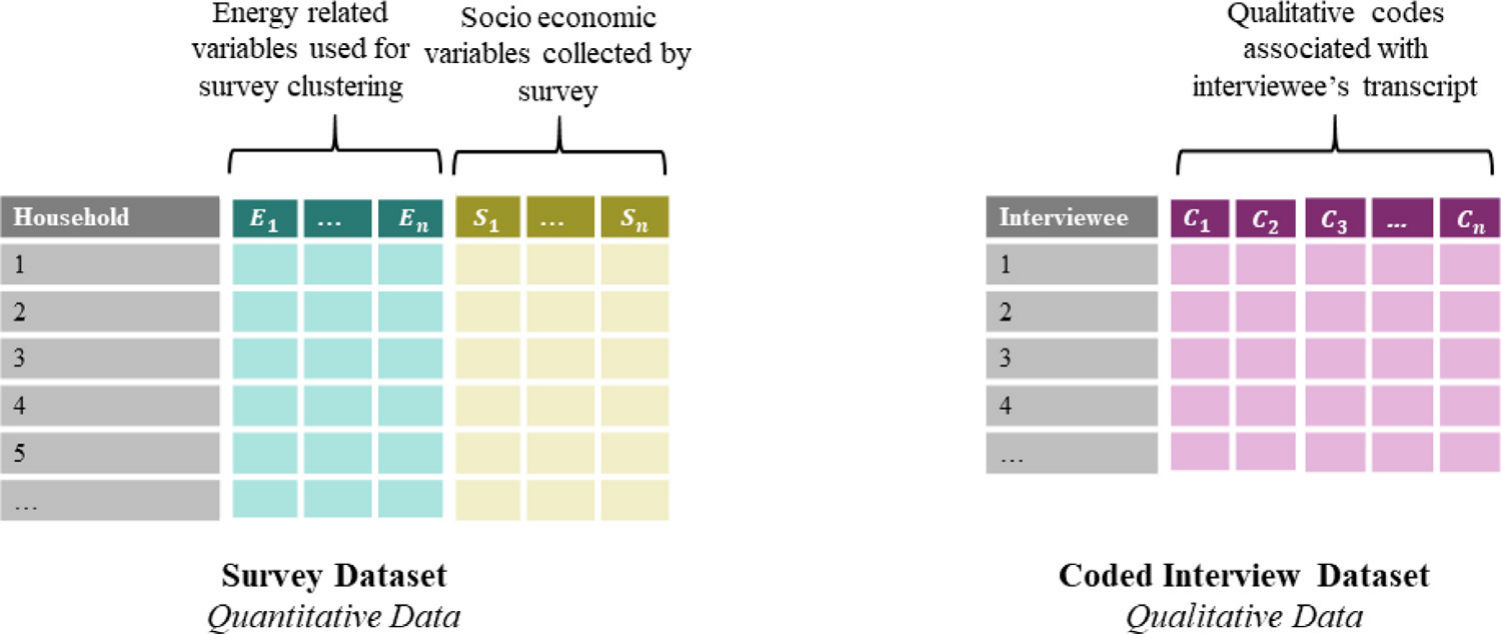
Schematic of mixed data structure. Two distinct datasets are required: one contains qualitative household level survey data with a mix of energy and socio-economic variables, the other dataset consists of a table of codings applied to the transcripts of interviewed households.

The first clustering step involves a separate cluster analysis of the qualitative interview data and quantitative survey data to identify common groups or communities amongst respondents on the basis of their energy use habits, decisions, and socio-economic and cultural circumstances. Hierarchical clustering methods were used for first step analyses, although slightly different approaches were required given the difference in data types. Fig. 2 shows the structure of the survey and interview datasets. The survey dataset contains a wide set of socio-economic and energy use variables, although only energy use variables will be used for clustering with the former used to characterise clusters. A conventional agglomerative hierarchical clustering method is used for community detection in the quantitative survey data. A grounded theory approach [23] is used to analyse the interview data. Codification of the transcripts provides data for a graph-based correlation clustering analysis, as shown in Fig. 2 the qualitative dataset for this analysis takes the form of a table of interview codes.

#### Quantitative survey clustering analysis

##### Variable selection & engineering

Variable selection is carried out to single out relevant variables and address multi-collinearity in the dataset which can make it difficult to identify relevant variables and quantify their effect. Correlation coefficients are used to select variables which have a significant correlation with clean fuel use. A Farrer-Glauber test [14] is used to identify multi-collinearity and where variables have a causal relationship, the less relevant variable is excluded from the dataset. Some variables from the survey data may be combined or engineered from the data set to facilitate clustering analysis and reduce the number of binary variables in the dataset. In the case study for Bangalore this involved creating compound appliance ownership variables where appliances were grouped by type (IT & Communication, Cooking, Heating & Cooling), and variables were created to denote the percentage of each type owned. Table 1 shows the energy use related variables selected from the survey data in the Bangalore case study.

**Table 1:** Selected energy use variables from survey dataset for use in Bangalore case study clustering, with reference to column in sample dataset.

Variable	Unit/Type	Name in sample dataset
Monthly LPG Use	*kWh/month*	*lpg_kwh*
Monthly Electricity Use	*kWh/month*	*electric_kwh*
Monthly Kerosene Use	*kWh/month*	*kerosene_kwh*
Daily Electricity Availability	*Hours/day*	*electricity_hours*
Hours of Cooking	*Hours/day*	*cooking_hours*
Hours of Lighting	*Hours/day*	*lighting_hours*
IT Appliance Ownership	*%*	*it_appliances*
Cooking Appliance Ownership	*%*	*cooking_appliances*
Government LPG Support Awareness	*Binary*	*programme_awareness*
Cooking Location	*Categorical*	*cooking_location*

##### Hierarchical clustering

The primary clustering of the survey data is performed using hierarchical clustering. In the Bangalore case study agglomerative hierarchical clustering was found to produce more balanced clustering, although it is recommended to try both agglomerative and divisive methods to determine which produces a more balanced set of clusters with a clearer optimal number of clusters. The Gower distance measure should be used to enable inclusion of categorical variables [26], and Ward's linkage criterion is used for clustering. Ward's linkage criterion identifies clusters to merge based on the lowest lack-of-fit sum of squares [50].

**Fig. 3:**
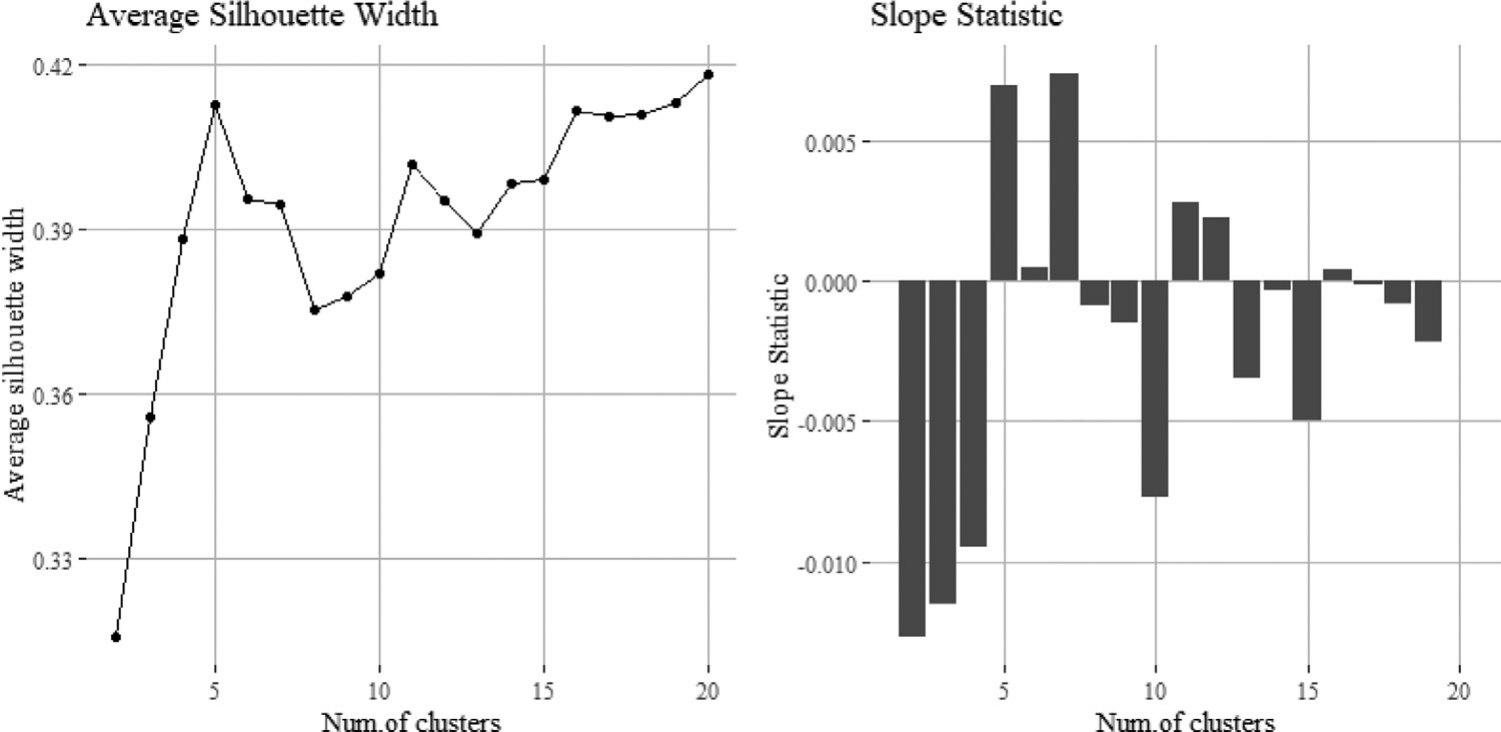
Example of use of Silhouette Width and Slope Statistic to determine optimal number of clusters for a sample of households in Bangalore. Note how while there are maxima on the silhouette width plot at 5 and 16 the slope statistic indicates 5 as preferable.

To determine the correct number of clusters the silhouette width method [43] is used alongside the elbow method and Fujita et al.’s [19] slope statistic. While there are several other methods such as the gap statistic [48] or the CH index [5]. Fujita et al. [19] found the combination of the silhouette and slope statistic to be relatively simple and effective when used together to identify the optimum number of clusters. The key is to use more than one method or approach given the overlapping nature of clusters when using high-dimensional data, as any one method may be ambiguous as to the optimal number. The slope statistic is given by Eq. (1) which states the optimum number of clusters $\hat{k}$ is given where a large silhouette value is given for k clusters, followed by a significant decrease in the silhouette value for the subsequent k+1 clusters. This was carried out using the base packages in *R* as well as the 'dendextend' and 'fpc' packages [20,28]. An example of this cluster number determination for the case study of Bangalore is shown in Fig. 3 which shows the average silhouette width having a first local maximum at *k* = 5, supported by a high positive value of slope statistic indicating 5 as an optimal number of clusters.

(1) $$\hat{k}=\arg\max\left(-\left[s\left(k+1\right)-s\left(k\right)\right]s{\left(k\right)}^{p}\right)$$

where k is the is the current cluster

$\hat{k}$ is the optimal number of clusters
s is the silhouette value
p is a positive integer tuned to weight importance of either the subsequent slope (small *p*) or the silhouette value of the current k. In our analysis this is set to 1.

#### Qualitative interview analysis

##### Qualitative data analysis – interview coding

The analysis of the qualitative interview data uses a grounded theory approach to qualitative data analysis, which as defined by Glaser and Strauss [23] is concerned with the systematic discovery of theory from data that both fits real world scenarios and can be easily understood by stakeholders. This approach is particularly relevant to the challenge of understanding residential energy use and transitions, because as explained by Corbin and Strauss [8] it provides a common language of concepts which stakeholders can engage with to address energy access issues, and this is key to an designing effective solutions for inclusive energy transition.

A codified approach to analysing qualitative data in a grounded approach is important to convey credibility and understand how narratives and pathways are derived from the data [23], and coding of the interview transcripts is used to quantify key discussion points and content. This is a form of quantizing as described by Sandelowski [46] and involves reducing interview transcripts into variables that can be associated with each interviewee. This will allow for the combined clustering of the qualitative and quantitative data through the second step clustering.

A first run of coding is sometimes referred to as open coding and is carried out to identify concepts from the data [8], using line-by-line analysis. Following this initial provisional coding, the concepts identified are analysed to determine the categories that these concepts might fall under. Detailed codes are deduced from the open coding to form a list of second level codes, while common properties of certain concepts are used to help define broader first level codes. The transcripts are labeled with these first level codes indicating categories, and then a second run of coding is carried out on the interview transcripts by the team of researchers using the refined set of more detailed second level-codes to narrow in on a more specific categorisation of the coded section of the transcript [6].

The interviews were coded and analysed using the 'RQDA' package in *R* [31], which provided a graphical interface for the coding process and facilitated export of datasets to the *R* environment for analysis alongside the quantitative survey data. The coded interviews were peer-reviewed to eliminate bias of the individual researchers, and disagreement between coders was addressed by an additional round of coding assessment drawing on subject-specific expertise in line with recommendation from previous studies [6,36].

#### Correlation clustering

**Fig. 4:**
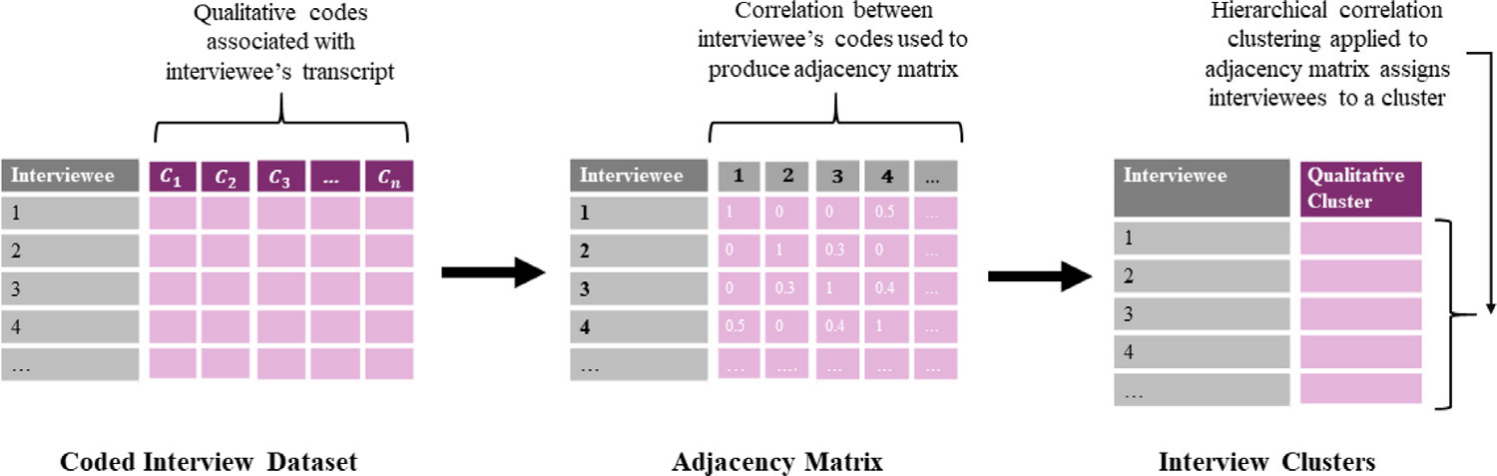
Schematic of interview coding clustering process. Coded interview table is transformed into an adjacency matric by calculating correlation between respondents. This adjacency matrix is then clustered to assign interviewees to respective clusters.

To identify different clusters amongst the interviewed households on the basis of transcript codings a correlation clustering approach is used. This approach was first introduced by Bansal et al. [2], and involves clustering a set of instances, in this case interviewed households using an adjacency matrix [42]. A schematic for this procedure is shown in Fig. 4, showing how the interview codings table is processed and clustered. To produce the adjacency matrix for the interview data a correlation matrix is calculated from the interview codings. The resulting adjacency matrix can be visualised as a graph of the links between the different respondents with proximity and thicker edges indicating similar topics discussed in the interview. Setting a correlation threshold for visualisation can make the resulting graph easier to interpret.

The adjacency matrix is clustered using a form of hierarchical clustering which known as fast greedy clustering. This detects communities within the graph by directly optimizing modularity, as explained by Clauset et al. [7]. In the case study of Bangalore the correlation threshold is set to at 0.3 such that any interviewee correlation below this is set to zero, increasing clarity in the graph by removing weak and negative links. All remaining positive non-zero values indicate a connection between two vertices. The definition of modularity used by this algorithm is shown in Eq. (2).

(2) $$Q=\;\frac{1}{2m}\sum_{vw}\left[A_{vw}-\frac{k_{v}k_{w}}{2m}\right]\delta \left(c_{v},c_{w}\right)$$

where Q is the modularity

m number of edges in the graph, $m=\frac{1}{2}\sum_{vw}A_{vw}$
v and w are a pair of vertices being considered
$A_{vw}$ is 1 where vertices v and w are connected, and 0 otherwise
$k_{v}$ the degree of vertex v defined as the number of edges incident upon it
δ is a function δ(i,j) which equals 1 when i=j, and 0 otherwise
$c_{v}$ is the community to which vertex v belongs

**Fig. 5:**
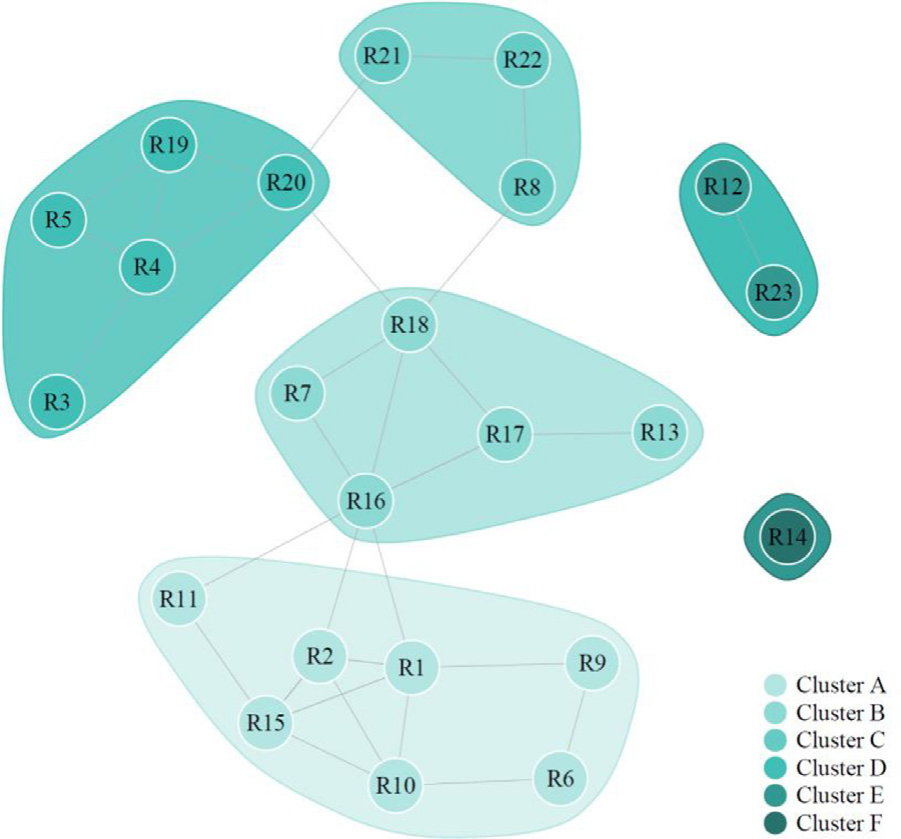
Network graph of communities detected amongst interviewees based on interview coding correlation. These groups form the qualitative interview clusters.

Unlike the clustering methods used on the quantitative survey data, modularity optimizing correlation clustering does not require specification of, or additional calculation to determine the optimal number of clusters. This analysis was implemented in *R* using the ‘igraph’ package [9], and the resulting clustered network of interviewees for the Bangalore case study are shown in Fig. 5. Notice how the linkages between clusters can help indicate clusters which may have some features in common or indicate members of cluster which may have commonalities with members of other clusters.

### Second step pathway identification

The second stage clustering analysis combines the information gained through the separate community detection of the qualitative interview and quantitative survey data respectively and identifies commonalities between these that characterise distinct energy transition pathways with socio-economic characteristics. This involves first matching interviewed households to a quantitative survey cluster before performing a secondary clustering of these households based on their quantitative and qualitative cluster membership.

#### Interviewee survey cluster matching

**Fig. 6:**
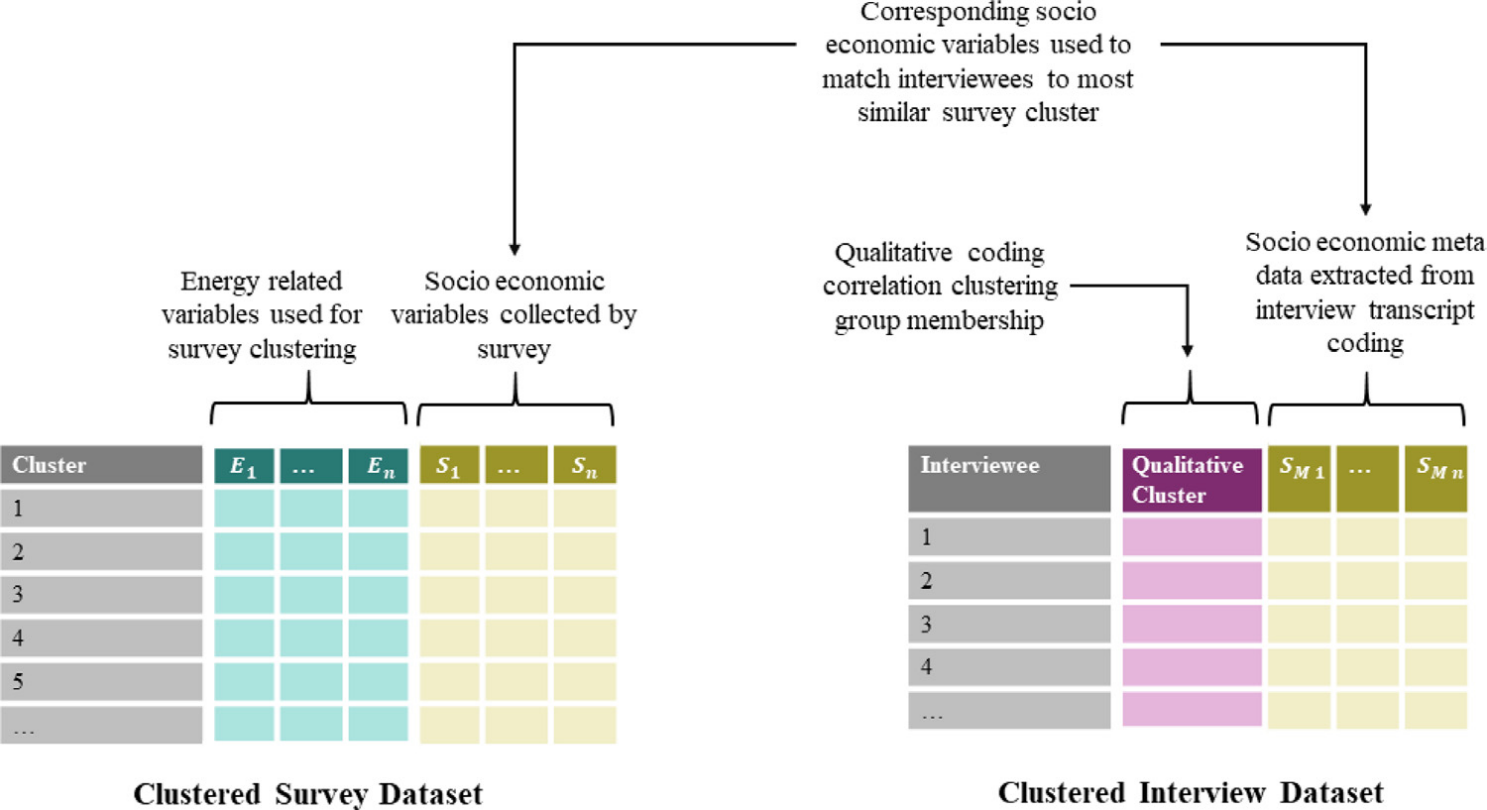
Schematic of interviewee survey cluster matching based on socio-economic variables.

The qualitative and quantitative data analysed in step one yields two sets of clusters defined by different features and variables. In order to map one set of clusters to the other, one set of clustered respondents must be matched to their closest clusters in the other dataset. To do this each of the interviewed households is matched to one of the survey clusters such that there are a set of households which have both an interview and survey cluster assigned. In theory this could be done the other way around, however it would require interviewing all the survey households and would be impractical for large sample sizes. To match the households to a cluster, common categorical variables need to be extracted from the interview transcript to create a metadata tag with household characteristics which can be compared to the survey cluster centroids. This is performed during the qualitative data coding described above, and the schematic in Fig. 6 shows how data is used for clustering compared to what is used for household matching. To ensure that these variables will be present in the interview transcripts and comparable to the survey data, specific prompt questions referring to these are included in the script for the semi-structured interviews. The interviewer can prompt a response on these variables if they are not brought up by the interviewee during the course of the interview.

Using these categorical matching tags each interviewee is assigned to the most similar survey cluster using Euclidean distance to measure similarity. This can accommodate any number of matching variables *n*, Eq. (3). The variables used to match the interviewees to a survey cluster are listed in the Table 2 and cluster centroid values used for distance measurement are based on mean values for each quantitative survey cluster. The variables used for matching survey and interview clusters include a combination of socio-economic variables and energy use variables such as primary cooking fuel, and presence of an electrical meter. This aims to ensure accurate matching even in cases where survey clusters cannot be distinguished based on the socioeconomic variables alone. In the Bangalore case study, the survey data was collected first, and matching variables were selected based on variables that displayed marked differences between clusters in the survey data.

(3) $$d=\sqrt{\sum_{i=1}^{n}{\left(x_{i}-y_{i}\right)}^{2}}$$

where d is the Euclidean distance

n is the number of dimensions,
x and y are a pair of points representing the
interviewee and the cluster centroid

**Table 2:** Table of variables used for interviewee to survey cluster matching.

Variable	Unit/Type
Time since Migration	*Years*
Income Frequency	*Categorical*
Primary Cooking Fuel: LPG	*Binary*
Primary Cooking Fuel: Kerosene	*Binary*
Primary Cooking Fuel: Biomass	*Binary*
Majority Religion	*Binary*
Legal Electricity Connection	*Binary*

A possible improvement to the matching process would be to include a continuous energy related variable such as fuel consumption to facilitate differentiation between nearest cluster. However, this would require asking a more numerical and detail-oriented question in the interview which could distract from encouraging interviewees to speak freely about their experience. It could also act as a leading question in that it could give respondents the incorrect impression that the interviewer is primarily concerned with expenditure and consumption, and so collection of such data in the interview should be carefully considered.

#### Cluster membership clustering

**Fig. 7:**
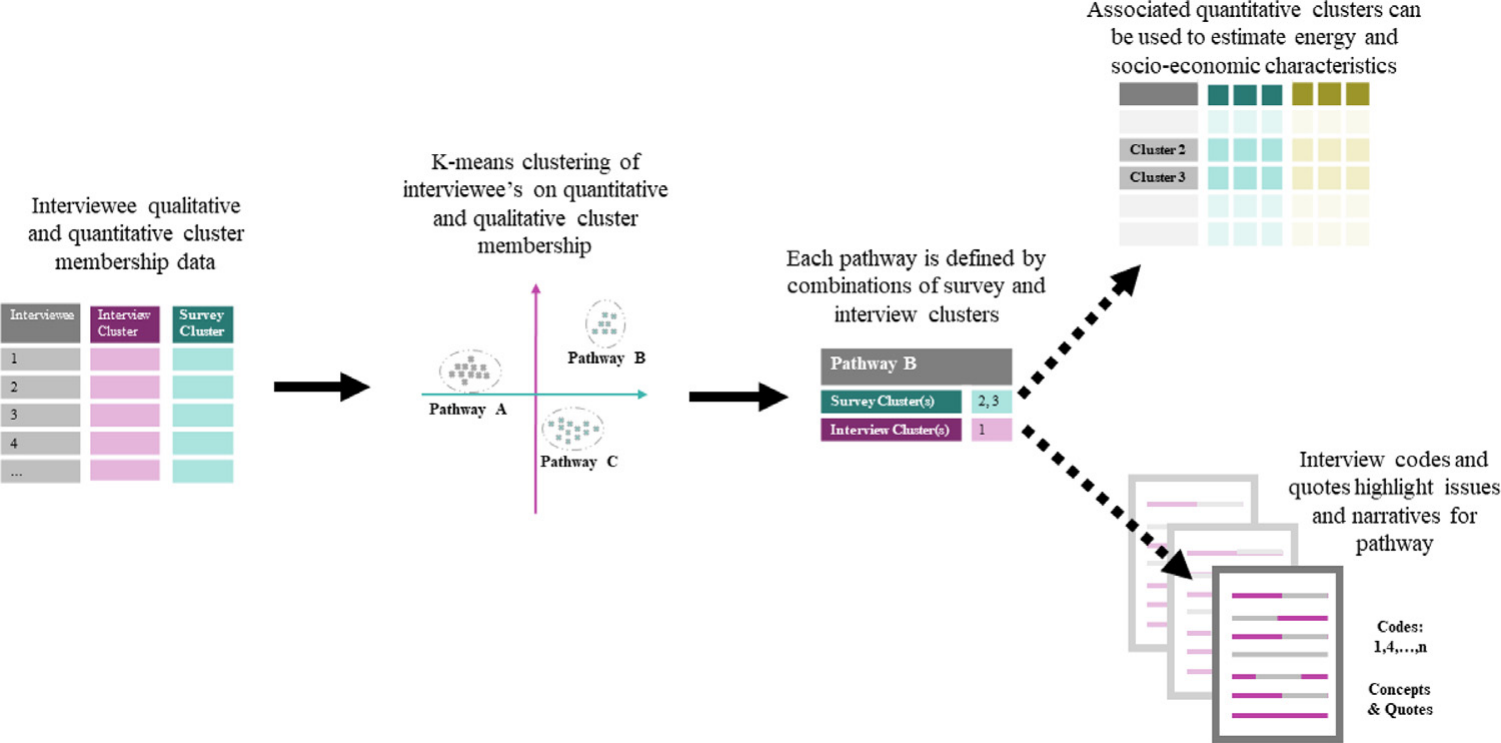
Schematic of second stage clustering and pathway characterisation. *K*-means clustering of the interview households using their qualitative and quantitative survey clusters as variables identifies pathway groups with unique pairs of.

Once assigned to a quantitative survey cluster each of the interview households will have membership of both a quantitative and qualitative cluster. The interview households are then clustered on their qualitative and quantitative cluster membership, that is to say the only variables used to cluster these households a second time are the quantitative and qualitative cluster numbers. A *k*-means clustering approach is used to perform this clustering – the same agglomerative hierarchical clustering used in the first step could also be used but given the tendency for these clusters of clusters to group into distinct non-overlapping and spherical clusters means the simpler *k*-means approach is well suited. Fig. 7 shows the process of second stage clustering and exemplifies how pathways can be characterised by drawing upon the quantitative variable ranges of the associated quantitative clusters, and codes, concepts, and quotes from the interviewees in the respective interview cluster which together can define a transition pathway and provide a narrative and common language of concepts for understanding the energy access challenges of the given pathway.

In order to determine the ideal number of clusters for the second stage clustering the elbow method is used alongside the silhouette width method and slope statistic, although given the low number of variables assessing a scatter plot of the cluster membership of these households will also show the clear divisions between groups of households. By using descriptive statistics for each of the constituent quantitative clusters a summary of weighted mean values and ranges for energy use patterns and socio-economic characteristics can be calculated for each pathway. This information is augmented by linking these to the key narratives identified in the associated qualitative interview cluster. This is particularly important as it provides not only a data driven description of the circumstances of households on each pathway, but also offers insight into the practices and decision-making rationale associated with households that fit these. The results of such an analysis can be particularly useful for local policy making as they combine data useful for technical insight with explanations for these observations derived from household level data, providing a common language which all stakeholders can understand and engage with.

## Example outputs

**Fig. 8:**
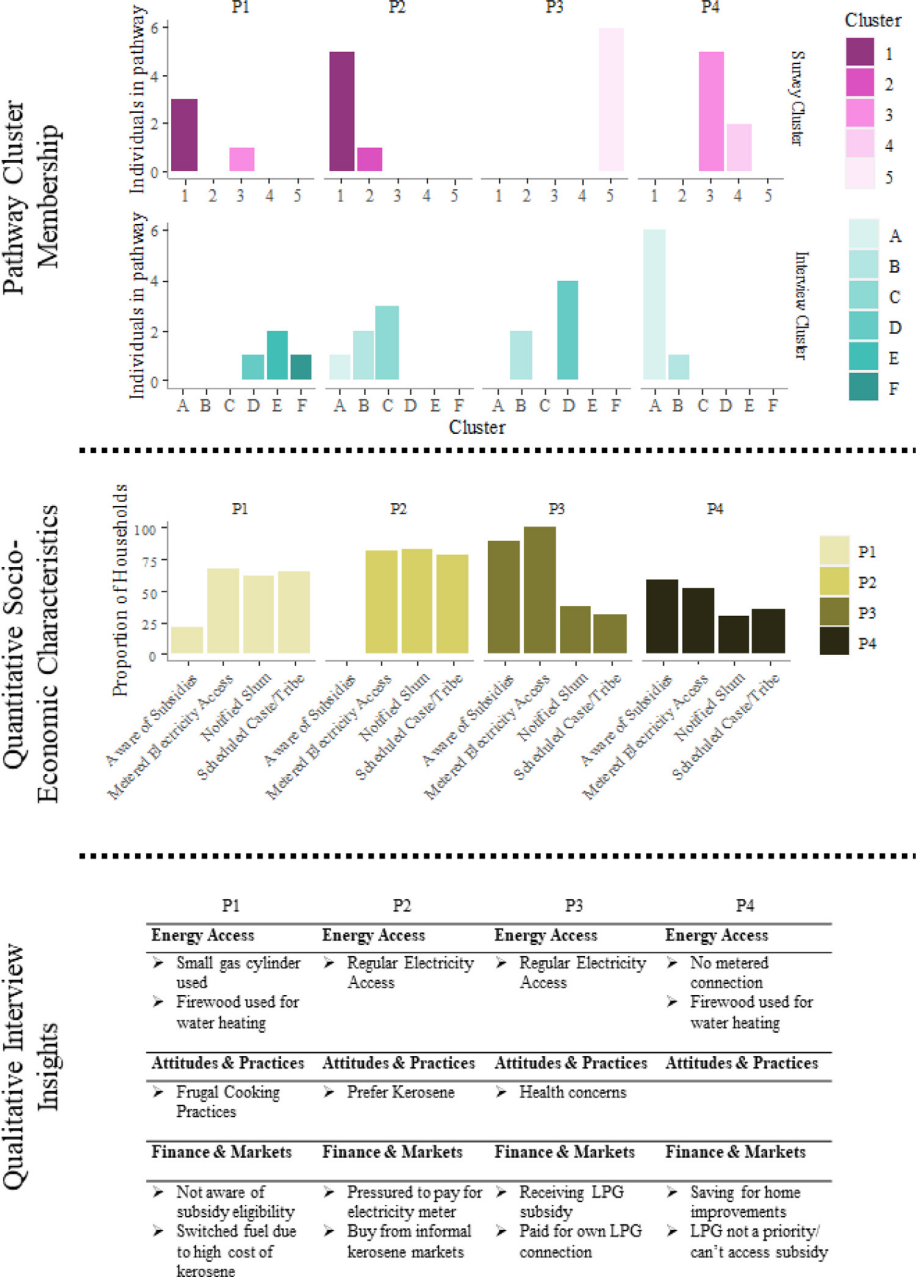
Graphic showing pathway cluster membership, associated socio-economic characteristics, and key qualitative interview data codes for each pathway in the example of low-income households in Bangalore.

In the Bangalore case study there are four clear pathways, and each cluster is composed of a distinct combination of quantitative and qualitative clusters. Fig. 8 details the breakdown of cluster membership for each of the four identified pathways, illustrating how using the cluster membership weighted socio-economic characteristics can be calculated, and the most common interview coding and selected quotes can be identified for each of the four pathways. In this case study households all have homogenous monthly expenditures, yet exhibit differences in awareness of subsidies and access to metered electricity (as opposed to illegally tapped electricity or non-grid sources). Households on these pathways are at different stages in transition to clean cooking fuel, with those on pathway P3 using clean fuels regularly, and those on pathway P4 hardly using any at all, and P1 and P2 somewhere in between these.

The simple and clear matching of interview clusters and survey clusters is an important feature, which can offer additional information. Distinguishing interview and survey clusters in each pathway can identify cases where a group of households with homogenous socio-economic characteristics can face distinctly different problems identified through the interviews. For example, pathways P1 and P2 both feature households from survey cluster 1, but pathway P1 identifies issues of high cost of kerosene driving change in cooking fuel and use of frugal cooking practices, whereas pathway P2 indicates household have a preference for kerosene and may seek it out from informal black market sources. This is important for supporting design of policy and interventions as often criteria for access eligibility for policies are based on socio-economic criteria. The matching with interview clusters allows for leveraging the explanatory power of qualitative data analysis methods used in social science research to offer narratives for observed energy use trends in the quantitative survey data.

This approach has some shortcomings when compared to recent methods for clustering mixed data. For example, the use of weighting schemes to adjust the contribution to cluster determination of each data type (numerical, textual, etc.) are an important consideration in clustering mixed data. The method presented in this paper does not calculate weightings for the different datatypes but rather applies equal weight to the survey and interview data, indeed such weights can be difficult to choose optimally [16]. Additionally, this method uses the commonly used Gower distance for clustering numerical and a simple Euclidean distance measure for cluster matching. However while popular and easy to implement with ready made functions, as pointed out by Foss et al. [16] the Gower distance measure is not without its problems, and selection of appropriate distance measures is a major consideration in mixed data clustering and dependent on the context of the data [29].

Two recently proposed methods for clustering mixed data are the Fuzzy C-Medoids clustering model [13] and KAMILA [15], could be used to cluster all the mixed data in the first instance, calculating appropriate weights on an objective basis and using distance measures more appropriate across the range of datatypes. However, a key advantage of the approach presented in this paper with respect to energy research applications is that it preserves the information gained from the separate quantitative and qualitative analysis in the first stage which can leverage discipline specific knowledge that can provide important insights for stakeholders as exemplified above. In addition, the simplicity of this method can facilitate collaboration between disciplines on energy research. The matching of interview and survey clusters draws a clear link between findings of qualitative analysis of the interviews and the quantitative analysis of the survey data and how they contribute to the final pathways. Such links would not be obvious if using mixed data clustering methods to cluster all the data in the first instance. Ensuring common understanding and sharing of data across disciplines and methodological divides is key to facilitating interdisciplinary energy research.

## Conclusions

This paper proposes a mixed methods approach for identifying residential energy transition pathways, which integrates quantitative and qualitative data and methods. Using clustering methods in a two stage analysis this method first analyses qualitative and quantitative data, identifying clusters on the basis of these different datatypes individually. A second stage of clustering identifies links between these qualitative and quantitative clusters and enables inference of energy transition pathways followed by low-income urban households defined by both quantitative characteristics and qualitative narratives. This clear link between pathways and associated qualitative and quantitative clusters can offer additional information by identifying cases where different energy access problems might not be apparent based on socio-economic characteristics alone, or where different clusters defined by socio-economic and energy use data might in fact face similar challenges identified through interviews. Importantly this approach also allows clear identification of how findings from qualitative and quantitative analysis in the first stage relate to identified pathways and energy transition problems second stage, which can facilitate interdisciplinary collaboration and comparison of data.

Further work could look at how other datatypes could be integrated, such as timeseries data on energy demand profiles. Other mixed data clustering methods could provide objective methods for weighting contributions from the multiple datatypes, and alterative distance measures could be used – however this would have to be integrated in such a manner that did not lose information gained from the qualitative data analysis, otherwise this would reduce the method to a purely quantitative analysis. The use of fuzzy clustering methods could also be explored, which would offer a measure of uncertainty in cluster assignments.

## Declaration of Competing Interest

The authors declare that they have no known competing financial interests or personal relationships that could have appeared to influence the work reported in this paper.

## Figures and Tables

**Table 3:** Overview of quantitative survey sections, data types collected and origin of questions.

Section	Heading	Question Source	Type of Data
1	Household identification	*Abridged from IHDS-II*	Socio-cultural indicators
2	Household roster	*Abridged from IHDS-II*	Demographic indicators
3	Occupation and salary	*Abridged from IHDS-II*	Economic indicators
4	Education	*Abridged from IHDS-II*	Socio-economic indicators
5	Appliance ownership	*Expanded from IHDS-II*	Appliance ownership
6	Fuel use	*Developed from scratch*	Fuel use magnitude
7	Energy use habits	*Developed from scratch*	Energy use practice

**Table 4:** Table of Census City Ward Level Variables used for Ward Selection. Note the rank ordering indicates whether high values of this variable (ascending) received a high rank score, or low values (descending).

Variable	Rank Ordering
Access to Banking (%)	*Descending*
Home Ownership status: Rented (%)	*Ascending*
Primary Cooking Fuel: Kerosene (%)	*Ascending*
Primary Cooking Fuel: LPG (%)	*Descending*
Overall Asset Ownership (%)	*Descending*
Lighting Energy Source: Electricity (%)	*Descending*
Lighting Energy Source: Solar (%)	*Ascending*

**Table 5:** Table of required sample size for Bangalore case study given different margins of error in estimated mean LPG fuel use, at a confidence level of 95 %.

Margin of Error, *E*	Required Sample Size, *n*
1.9 kWh (1.0%)	5674
4.8 kWh (2.5%)	892
9.6 kWh (5.0%)	226
14.4 kWh (7.5%)	102
19.3 kWh (10.0%)	58
24.1 kWh (12.5%)	39

**Table 6:** Structure of semi-structured interview based on four key topics, the table indicates the issues which the interviewer sought to discuss in connection to each topic.

Topic	Issues discussed
Energy consumption	Preference of cooking fuels, appliance usage, aspirations, knowledge of health impacts of fuels;
Financing	Expenditure and budgeting habits of households related to energy costs, frequency of replacement of LPG cylinders, access to formal/informal loans;
Social network	Participants’ involvement with community networks and ability to rely on the same for support, financial or otherwise (e.g., community lending or savings clubs or sharing information regarding schemes);
Political network	Participants’ relationship with existing local government, involvement with political associations, experiences in community mobilisation;

## Additional information

### Data collection protocol

The mixed method clustering requires a dataset with the features detailed in Fig. 2 which contains survey data from a sample of low-income households across an urban region and semi-structured interviews with a smaller sample of households from the same region. The semi-structured interviews must encompass the typologies of households identified in the survey data. Any dataset that satisfies these requirements could be used in the mixed method pathway clustering. However, if designing a study to include data collection this additional information provides details on the protocol for survey and interview design and sampling used in the case study.

#### Survey design

##### Pre-testing of questions

Pre-testing of the survey instrument on a small group of respondents can identify questions that are difficult to understand or are interpreted in a manner other than intended by the researcher and allow for the above criteria to be assessed before widespread data collection. Typically a small group of 15–25 respondents will be used for pre-testing with a debriefing session following the survey to understand respondent's experience [4,37]. More recently, variations on this approach have been introduced such as behaviour coding [17] in which an observer monitors the pre-test interview and takes note of problems in interpretation, re-reading of questions, and misunderstandings and relevant questions are reviewed. Another approach - which was employed for this case study data collection - is cognitive pretesting where respondents "think aloud" while answering questions to allow interviewers to understand how the question is being interpreted [3,10].

##### Design of survey instrument

An underlying criteria for the design of the survey instrument is to ensure the potential for compatibility and cross-referencing with the existing IHDS and to a lesser extent other NSS and Census data. To allow for this, a selection of questions characterising the household in terms of household composition, dwelling type, caste, religion, expenditure, and education were taken from the IHDS-II (2011) survey [11]. Table 1 details the structure of the survey in terms of composition of questions and data sought. The questions developed from scratch for this survey were geared at probing energy use behaviours. Table 3 summarises the sections of the survey questionnaire.

Survey enumerators were recruited from the local area and were fluent in the commonly used local languages and familiar with the areas being surveyed. A training session was held with the enumerators prior to data collection to ensure understanding of the questionnaire, and to address potentially problematic questions or missing answer options. The survey was encoded as an ODK XForm to enable survey responses to be directly recorded in a digital format through an app ensuring correct encoding. Enumerators completed surveys on tablet computers, this ensured that data was immediately encoded, tabulated, and uploaded to the server reducing the risk of any mistakes in transcribing from paper surveys.

#### Survey sample area

The selection of sample area is important as cities in India can exhibit substantial spatial inequality and certain city wards will have a far greater proportion of low-income households than others. The 2011 Indian census data [24] includes city-ward level data on a limited number of socio-economic variables, including primary cooking fuel choice, lighting fuel choice, and ownership of a group of electronic appliances (TV, mobile phone, radio, scooter). Table 4 shows the variables in the census ward-level data used for ward selection. The selection of wards was performed using a rank score on the variables of interest, to identify wards with socio-economic features which suggest a high proportion of low-income energy poor households. Eqn. (A1) gives the rank score used for shortlisting of wards, and final selection of wards was based on these rank scores and local knowledge of enumerators familiar with logistical and political characteristics of the local area.

(A1) $$Score=\;\sum_{i=1}^{n}\frac{\left(W+1\right)-\;x_{i}}{W}$$

where n is the number of variables used for ward selection,

W is the total number of wards,
$x_{i}$ is the rank of the given ward for the ith variable.

A balance must often be struck between desired data for the survey and the logistical and political practicalities of conducting surveys in specific wards or communities within a city. In Bangalore case study, seven wards were selected using this approach. These wards are of interest either for low access to finance or home ownership, or for high use of alternative cooking fuels which imply that households are at a ’tipping point’ having to choose between two prevalent options. The use of a mix of cooking fuel also suggests ongoing change and that in the seven years since the census there will have been more adopters of the modern fuel. Importantly, such recent adopter households will be more likely to remember reasons and drivers behind their adoption of the new fuel as well as recall changes in behaviour because of said adoption.

#### Sample sizing

The survey comprises a range of quantitative questions whose purpose is to determine population means, as well as qualitative questions with categorisation which will not follow a normal distribution. The selection of sample size for qualitative surveys cannot be obtained purely by calculation and often relies on precedent and best practice [33,35], although some studies have sought to employ quantitative statistical test power measures of sample size based on theme prevalence [18]. Others have pointed out a trade-off between higher information power of small samples and greater statistical power of larger sample sizes [39].

The sample can be definitively sized for the key quantitative data, in this case magnitude of fuel use, and in practice this is likely to be the limiting sample size criteria. There are several approaches that can be taken, one method is to define the width of confidence interval for the mean of the parameter of interest and calculate the sample size required to deliver this, or the power of a test hypothesis on the parameters of interest can be calculated to determine the minimum sample size to attain a certain power of test value [38]. There is a Bayesian approach which is well suited in cases where there is a prior distribution of the desired parameter, and can use this in place of making a guess [45].

In the case study of Bangalore, which is likely similar to most large Indian cities, while there are prior distributions available they are several years out of date and thus using an estimate for the expected mean value offers a sensible method for determining sample size. The aim is to have a representative sample within each urban district or community surveyed. For the purposes of inferring key transition pathways and providing supporting quantitative data for these pathways a desired accuracy for mean fuel use estimates of +/- 10% at a 95% confidence level was chosen, and assuming that energy use is approximately normally distributed the sample size can be calculated using Eqn. (A2).

(A2) $$n=\frac{{\left(Z_{\alpha /2}\right)}^{2}\sigma^{2}}{E^{2}}$$

where n is sample size,

σ standard deviation,
$Z_{\alpha /2}$ value of Z providing an area of α/2 in upper tail of normal distribution,
E margin of error

Obviously one of the problematic values in this calculation is the standard deviation of the expected data. Anderson and David [1] discusses several possible approaches, including using the standard deviation from a previous study, the standard deviation from a pilot study, or a ‘best guess’ approach which involves estimating upper and lower bounds of the population. Given that there is existing data for Indian cities (albeit out-dated, from the IHDS), the initial sample sizing calculation has been based off the values from this. Table 5 below shows the indicated sample size for different magnitude of error on the mean LPG monthly use. In the case of the Bangalore case study a ward sample size of 60 (rounded up from 58) was selected, and the 7 wards identified to survey made for a total sample size of 420. A useful check can be done using early data from the survey collection to assess suitability of sample size and agreement with the prior mean value used in this calculation.

#### Interview design

The qualitative dataset consists of in-depth semi-structured interviews with a sample of households from the same geographic area selected for the survey. This form of data collection is common in the social sciences for studying issues ranging from urban inequality to gender studies [6]. The anonymity of surveyed households were safeguarded by not collecting their addresses. In other words, it is not possible to return to the specific households covered by the quantitative surveys. Instead, from the seven wards where the survey was conducted, a purposive sample informed by the different types of households identified in the preliminary survey analysis were interviewed.

As previously discussed, sizing of samples for qualitative studies is typically based on previous experience and best practice. The advice of Guest et al. [27], and the precedent set within the field [21,34] that more than 12 interviews provides a reasonably high probability of identifying key issues was followed, and 23 interviews were carried out (24 were although one respondent declined to continue interview). Selection of interviewees was targeted to feature a higher proportion of households representing outlier clusters in the survey analysis while ensuring representation of all cluster types in the survey cluster analysis which is detailed below. Expert knowledge from the survey enumerators helped inform the selection of these households.

The 30 min semi-structured interviews allowed flexibility in identifying and discussing issues important to participants. The interview was structured to cover four broad topics, namely household energy consumption preferences and practices, finances, social networks/community, and political networks/interactions. Table 6 details the issues the interviewer sought to discuss under each of these topics. The interviews were conducted in Kannada and Tamil and transcribed to English and stored as digital text files for coding and further analysis.

*Ethics*

Collection of survey and interview data in our Bangalore study received ethical approval. As part of compliance with this participants were provided with information regarding the research before deciding to consent to the interview. Participants were informed that they could refuse at any time to be surveyed, and that no personal data would be shared. Only fully anonymised and processed datasets are made available, and this dataset is included with the sample *R* code in the supplementary data to this article. Full interview transcripts could be used to identify individuals, therefore only anonymised excerpts can be published.

### A note on alternative clustering methods

While *k*-means clustering is used for the second step clustering, hierarchical clustering methods were used for the first stage qualitative and quantitative clustering. In the case of the quantitative clustering *k*-means clustering was used initially, however it did not produce as clear a division of clusters. This can be partly attributed to the large number of variables used as well as the non-spherical shape of clusters. The use of hierarchical clustering has an added benefit that interpretation of the dendrogram alongside the silhouette width plot can make it easier to assess the optimal number of clusters in cases where the silhouette width method indicates two similarly optimal numbers of clusters.
